# Estimation of Health and Economic Benefits of Commercial Peanut Immunotherapy Products: A Cost-effectiveness Analysis

**DOI:** 10.1001/jamanetworkopen.2019.3242

**Published:** 2019-05-03

**Authors:** Marcus Shaker, Matthew Greenhawt

**Affiliations:** 1 Dartmouth-Hitchcock Medical Center, Section of Allergy and Immunology, Lebanon, New Hampshire; 2 Dartmouth Geisel School of Medicine, Hanover, New Hampshire; 3 Children’s Hospital Colorado, University of Colorado School of Medicine, Section of Allergy and Immunology, Food Challenge and Research Unit, Aurora

## Abstract

**Importance:**

Commercial epicutaneous peanut immunotherapy (EPIT) and peanut oral immunotherapy (POIT) may offer significant quality-of-life improvements for patients with peanut allergy, but the cost-effectiveness of commercial peanut immunotherapies is uncharacterized.

**Objective:**

To evaluate critical inputs associated with the cost-effectiveness of EPIT and POIT from a societal perspective.

**Design, Setting, and Participants:**

Economic evaluation in which microsimulations with Markov modeling were performed evaluating virtual children aged 4 years over an 80-year time horizon. The base-case costs included a caregiver-reported willingness to pay of $3839 annually for safe and effective food allergy treatment. Estimates of predictive biomarkers or oral challenges were incorporated after the first year of therapy with additional analyses of immunotherapy risk reduction of anaphylaxis and probability of sustained unresponsiveness (SU) to peanut after 4 years.

**Exposures:**

Children received EPIT, POIT, or no immunotherapy treatment (n = 10 000 per treatment strategy).

**Main Outcomes and Measures:**

Rates of therapy-associated adverse reactions and quality-of-life improvements associated with changes in eliciting or tolerated peanut doses were modeled along with quality-adjusted life-years (QALYs), anaphylaxis, therapy-associated anaphylaxis, and fatalities.

**Results:**

In the base-case analysis without SU to peanut, the EPIT strategy cost less than POIT (mean [SD] cost, $154 662 [$46 716] vs $163 524 [$56 800]) and had fewer total episodes of anaphylaxis (mean [SD], 1.33 [1.55] vs 3.83 [5.02] episodes) and fewer episodes of therapy-associated anaphylaxis (mean [SD], 0.62 [1.30] vs 3.10 [4.94] episodes) but had lower QALY accumulation (mean [SD], 26.932 [2.241] vs 26.945 [2.320] QALYs). The incremental cost-effectiveness ratio was $216 061 for EPIT and $255 431 for POIT. Models were sensitive to therapy cost, SU rates, health state utility, and risk reduction of anaphylaxis. With health state utility sensitivity analyses, the ceiling value-based cost (willingness-to-pay threshold $100 000/QALY) was between $1568 and $6568 for EPIT and between $1235 and $5235 for POIT. If high rates of SU to peanut can be achieved in longer-term models, EPIT and POIT could produce savings in terms of both cost and QALY.

**Conclusions and Relevance:**

In this simulated analysis, findings showed that EPIT and POIT may be cost-effective under some assumptions. Further research is needed to understand the degree of health state utility improvement associated with each therapy, degree of protection against anaphylaxis, and rates of SU.

## Key Points

**Question:**

Epicutaneous peanut immunotherapy and peanut oral immunotherapy may be approved in 2019 or 2020, but what variables will determine if these therapies will be cost-effective?

**Findings:**

This economic evaluation estimated a ceiling value-based cost (willingness-to-pay threshold $100 000 per quality-adjusted life-year) between $1568 and $6568 for epicutaneous peanut immunotherapy and between $1235 and $5235 for peanut oral immunotherapy. Cost-effectiveness of both therapies was sensitive to rates of sustained unresponsiveness, improvements in health state utility, and risk reduction of anaphylaxis.

**Meaning:**

Cost-effectiveness of epicutaneous peanut immunotherapy and peanut oral immunotherapy will be heavily influenced by their probability of sustained unresponsiveness, improvement in quality of life, risk reduction of anaphylaxis, and cost to patient.

## Teaser

This economic evaluation uses a simulation to calculate the cost-effectiveness of epicutaneous peanut immunotherapy and peanut oral immunotherapy under differing assumptions.

## Introduction

Peanut immunotherapy is poised to offer a significant shift in paradigms of care for patients with peanut allergy, and both oral and epicutaneous forms of therapy may be approved in the coming years.^1,2^ These new products may include a commercial peanut oral immunotherapy (POIT) treatment (AR101; Aimmune Therapeutics) as well as an epicutaneous peanut immunotherapy (EPIT) patch (Viaskin peanut; DBV Technologies). Neither therapy is curative (ie, creates a lasting tolerance allowing ad libitum peanut consumption), although both may offer some increase in the amount of peanut an individual can tolerate before reacting (or the amount at which a reaction will occur) of between 300 and 1000 mg of peanut protein (estimated to reduce the risk of reaction from accidental exposure from products with precautionary labeling by 95% to 99% and, at the upper ranges, protect from a small bite of a peanut-containing product) as well as some possible attenuation of the severity of a reaction on peanut exposure.^3,4,5,6^ However, patients treated with these therapies must still follow strict peanut avoidance and carry emergency medications. How these therapies may ultimately be used in the treatment of peanut allergy is incompletely understood.^7^ Both therapies have risks of adverse events, including anaphylaxis related to the treatment itself, although the degree to which patients may accept this as a trade-off is unknown.^8^ To better understand the potential health benefits and economic outcomes (eg, relative costs and value) of these commercial food allergy therapies, we performed Markov modeling with microsimulation and cost-effectiveness analysis.

## Methods

Per the Colorado Multiple Institutional Review Board, this study did not qualify as human subjects research and therefore did not require review or an exemption from review. The analysis followed the Consolidated Health Economic Evaluation Reporting Standards (CHEERS) reporting guideline.^9^

### Decision Model and Strategies Compared

A decision tree (eFigure 1 in the Supplement) was constructed using TreeAge Pro 2018 (TreeAge Software, Inc) to evaluate cost-effectiveness of peanut immunotherapies in children undergoing immunotherapy compared with children who receive no immunotherapy treatment and follow strict peanut avoidance with carriage of self-injectable epinephrine, which is the current standard of care.^10^ We compared groups of children with peanut allergy from a societal perspective (eg, public health perspective inclusive of patient and societal considerations such as direct medical and nonmedical costs, indirect costs, and intangible aspects) who received EPIT, POIT (in the form of the AR101 CODIT [Characterized Oral Desensitization Immunotherapy] commercial roasted peanut flour immunotherapy), and no immunotherapy treatment. Model start age was 4 years, which is the youngest age studied in the phase 3 trials for both products.^3,5^ All participants experienced probabilities and costs of peanut allergy. Participants receiving immunotherapy treatments also experienced probabilities associated with either EPIT or POIT while accumulating health state utility benefits associated with these therapies. Immunotherapy risks and benefits were applied with iterative dichotomous outcomes under unique probabilities and costs associated with each particular therapy. Microsimulations (cycle length, 1 year) were used (10 000 participants per treatment group) over an extended time horizon (80 cycles) to evaluate costs and outcomes in a Markov model. Microsimulation was used in the base case to evaluate all rates of anaphylaxis, therapy-associated anaphylaxis, and food allergy fatalities as well as to provide a measure of uncertainty resulting from rare fatal anaphylaxis. Because Markov models allow transitions between health states, the simulation incorporated the benefit of perceived risk reduction on quality of life (QoL) for children with peanut allergy while incorporating risks of each therapy and rate of discontinuation due to adverse effects, nonadherence, or treatment failure. Microsimulations were performed in the base-case analysis with trackers for all food allergy–associated fatalities, all food-associated anaphylaxis, and therapy-associated anaphylaxis. We consulted recently published reviews on peanut allergy and food allergy therapy and a recently published systematic review on food immunotherapy for reference inputs.^10,11,12,13,14,15,16,17,18,19,20^

### Health State Utilities

Health state utilities reflected quantitative patient-perceived value of peanut immunotherapy protection under conditions of risk. Baseline utility values for living with food allergy as well as disutility (utility toll) resulting from a severe allergic reaction were incorporated from the article by Carroll and Downs.^21^ We also included the benefit perceived by patients in QoL from protection offered by immunotherapy by applying the QoL improvement reported by Anagnostou et al^22^ against the QoL reported in untreated food allergy by Venter et al.^23^ For this calculation, a 1.61-point mean improvement in Food Allergy Quality of Life Questionnaire scores reported in participants treated with POIT (who were able to consume 800 mg of peanut protein) was applied to the base health state Food Allergy Quality of Life Questionnaire impairment to derive a 5.7% reduction in health state disutility (−0.09) for every 100 mg of peanut protein buffer allowed in the diet of treated individuals. This calculation was then used to derive a baseline health state utility for participants treated with peanut immunotherapy.

### Immunotherapy Treatments

In addition to perceived benefit from peanut immunotherapy, we also modeled the adverse event rates of therapy. For EPIT, a rate of treatment-emergent adverse events was modeled at 59.7% of participants with a rate of anaphylaxis of 3.4% and treatment discontinuation of 1.7%.^5^ The rate of anaphylaxis (described in the protocol as “systemic hypersensitivity”) for POIT was 14.2% (20.4% discontinuation rate, total 98.7% adverse event rate).^3^ Health state disutility toll was applied to treatment-induced anaphylaxis (−0.09 per occurrence).^21,24^

All participants were evaluated after the first year of therapy for response using a 1-time assessment of a predictive biomarker or oral challenge. Discontinuation rates were applied in the first year and in subsequent years it was assumed responders continued to adhere to the prescribed therapy. For EPIT, the peanut IgG4 to IgE ratio at the end of year 1 was evaluated, and patients reaching a level of 20.4 mg/L, shown to have an 80% positive predictive value for an eliciting dose of at least 300 mg of peanut protein, continued therapy (49.5% of participants at the end of 12 months of EPIT achieved this level, and in the simulation qualified to continue EPIT).^5,25^ For POIT, 67.2% of participants qualified as responders.^3^ To account for uncertainty in use of biomarkers, additional analyses evaluated models using supervised oral challenge after 1 year of therapy.

Models also evaluated rates of 4-year sustained unresponsiveness (SU; defined as ability to discontinue daily therapy for a period of time and then consume peanut without reacting)^7^ in 25%, 50%, and 75% of participants for the remaining duration of the model, with participants reaching SU assumed to achieve peanut tolerance.

### Additional Clinical Variables

The base case modeled persistent peanut allergy, as spontaneous tolerance becomes less likely in older children, adolescents, and adults. Spontaneous peanut tolerance was evaluated in supplemental sensitivity analyses, assuming annual rate of natural tolerance acquisition through age 20 years of 22% (range, 15%-30%).^24,26,27,28^ Children who experienced anaphylaxis through accidental exposure^24,29^ or therapy were evaluated in the emergency department as per standard recommendations,^30^ with 35% of those with anaphylaxis undergoing hospitalization.^31^ The model included all-cause age-adjusted mortality as well as risks of food allergy–associated fatality.^12,15,19,32^

### Costs, Discounts, and Horizon

Costs associated with peanut allergy included epinephrine autoinjector preparedness; visits to primary care clinicians, nutritionists, and alternative health professionals; grocery costs; and job-related opportunity costs, reflective of a societal perspective.^14,16,18,33,34,35^ All costs were expressed in 2018 US dollars, and costs and quality-adjusted life-years (QALYs) were equally discounted at 3% per annum.^36^ Total costs of all forms of immunotherapy (including POIT-related build-up clinician visits) were based on the caregiver reported annual willingness-to-pay (WTP) thresholds for safe and effective food allergy treatment^33^ ($3839 per year) in the base-case analyses, with cost-effectiveness ceiling thresholds investigated through cohort and microsimulation sensitivity analyses.

Sensitivity analyses were performed on all variables, exploring costs up to $20 000 per hospitalization for anaphylaxis.^37^ Cost-effective care was defined at a WTP threshold of $100 000 per QALY.^38^ Model variables and assumptions are shown in Table 1 (and a glossary of terms used in this analysis appears in eTable 1 in the Supplement).

**Table 1.** Simulation Model Inputs

Variable	Model Reference (Range)	Source
US life tables	National Vital Statistics Reports, April 2017	Arias et al, 2017^32^
Food allergy fatality	Age 5-19 y: 3.25 per million person-years (95% CI, 1.73-6.10; sensitivity, 3.25-33.00); age ≥20 y: 1.81 per million person-years (95% CI, 0.94-3.45; sensitivity, 1.81-18.1)	Umasunthar et al, 2013^12^
Rate of accidental peanut exposure and symptoms in peanut-allergic persons	7%/y (sensitivity, 5%-45%)	Neuman-Sunshine et al, 2012^24^
Adverse events from therapy	EPIT: anaphylaxis, 3.4% (sensitivity, 1%-10%); all adverse events, 59.7% (sensitivity, 20%-85%) and POIT: anaphylaxis, 14.2% (sensitivity, 5%-25%); all adverse events, 98.7% (sensitivity, 45%-99%)	Vickery et al, 2018^3^; Fleischer et al, 2019^5^
Rate of emergency department visit for anaphylaxis in peanut-allergic persons	1%/y (sensitivity, 0.5%-3.5%)	Neuman-Sunshine et al, 2012^24^; Capucilli et al, 2018^29^
Hospitalization following emergency department visit for anaphylaxis	35% (sensitivity, 5%-45%)	Robinson et al, 2017^31^
Pediatrician visits (mean incremental annual cost for food allergy diagnosis)	$100 (sensitivity, $94-$105)	Gupta et al, 2013^33^; Bureau of Labor Statistics^36^
Allergist visits for food allergy (mean incremental annual cost for food allergy diagnosis)	$149 (sensitivity, $140-$152)	Gupta et al, 2013^33^; Bureau of Labor Statistics^36^
Nutritionist visits for food allergy (per year)	$17 (sensitivity, $15-$18)	Gupta et al, 2013^33^; Bureau of Labor Statistics^36^
Alternative care professional visits for food allergy (per year)	$25 (sensitivity, $22-$27)	Gupta et al, 2013^33^; Bureau of Labor Statistics^36^
Incremental annual grocery costs (living with food allergy)	$310 (sensitivity, $290-$330)	Gupta et al, 2013^33^; Bureau of Labor Statistics^36^
Job-related opportunity costs from food allergy (per year)	$2597 (sensitivity, $0-$2697)	Gupta et al, 2013^33^; Bureau of Labor Statistics^36^
Personal epinephrine autoinjector	$715 (95% CI, $685-$743; sensitivity, $50-$1000)	Shaker et al, 2017^14^; Bureau of Labor Statistics^36^
sIgE/sIgG testing	$17 per test (sensitivity, $10-$234)	Healthcare Bluebook^34^
Oral food challenge	$121 (sensitivity, $115-$250)	Centers for Medicare & Medicaid Services physician fee schedule^35^
Hospitalization	$5899 (95% CI, $5732-$6066; sensitivity, $5000-$20 000)	Patel et al, 2011^37^; Bureau of Labor Statistics^36^
Emergency department visit	$691 (95% CI, $689-$693)	Patel et al, 2011^37^; Bureau of Labor Statistics^36^
Peanut immunotherapy willingness to pay	$3839 (sensitivity, $1200-$8000)	Gupta et al, 2013^33^; Bureau of Labor Statistics^36^
Start age	4 y (sensitivity, 2-8 y)	
Cycle length	1 y	
Annual discount rate	0.03 (sensitivity, 0-0.03)	
Negative health state influence for food allergy and food anaphylaxis	−0.09 (sensitivity, −0.02 to −0.11)	Carroll and Downs, 2009^21^
Negative health state influence for food allergy with EPIT and POIT	EPIT at 300 mg eliciting dose: −0.07 (sensitivity, −0.02 to −0.07); POIT at 600 mg tolerated dose: −0.06 (sensitivity, −0.02 to −0.06); POIT at 300 mg tolerated dose: −0.07 (sensitivity, −0.02 to −0.07)	Venter et al, 2015^23^; Anagnostou et al, 2014^22^

Abbreviations: EPIT, epicutaneous immunotherapy; POIT, peanut oral immunotherapy; sIgE, specific IgE; sIgG, specific IgG.

## Results

In this simulated analysis, compared with POIT, EPIT was associated with lower costs (mean [SD] cost, $154 662 [$46 716] vs $163 524 [$56 800]), fewer total episodes of anaphylaxis (mean [SD], 1.33 [1.55] vs 3.83 [5.02] episodes), and fewer episodes of therapy-associated anaphylaxis (mean [SD], 0.62 [1.30] vs 3.10 [4.94] episodes) but lower QALY accumulation (mean [SD], 26.932 [2.241] vs 26.945 [2.320] QALYs). Over the model horizon for no treatment (natural history model), costs (mean [SD] cost, $124 568 [$10 261]), QALYs (mean [SD], 26.792 [2.318] QALYs), and anaphylaxis episodes (mean [SD], 0.72 [0.85] episodes) were lower than for both treatments. Food allergy–associated fatalities were low among all groups as the model concluded (<0.001 occurrence). The net monetary benefit was greatest for EPIT vs POIT (mean [SD], $2 538 501 [$212 765] vs $2 530 961 [$220 589]). Neither EPIT nor POIT was cost-effective in the base model. Assuming an annual cost for each therapy of $3839 based on expressed caregiver WTP,^33^ the incremental cost-effectiveness ratio (ICER) was $216 061 for EPIT and $255 431 for POIT compared with no treatment (Table 2).

**Table 2.** Incremental Costs, Effectiveness, and ICER Compared With No Immunotherapy

Therapy	Mean (SD)						Simulations, No.	Incremental Cost, $	Incremental Effectiveness	ICER, $
	Cost, $	Effectiveness, QALYs	Net Monetary Benefit, $	All Anaphylaxis	Therapy-Associated Anaphylaxis	Food Allergy–Associated Fatality				
No immunotherapy	124 568 (10 261)	26.792 (2.318)	2 554 666 (221 717)	0.72 (0.85)	0	0.0001 (0.01)	10 000	NA	NA	NA
Epicutaneous immunotherapy	154 662 (46 716)	26.932 (2.241)	2 538 501 (212 765)	1.33 (1.55)	0.62 (1.3)	0.0002 (0.0141)	10 000	30 094	0.139	216 061
Peanut oral immunotherapy	163 524 (56 800)	26.945 (2.32)	2 530 961 (220 589)	3.83 (5.02)	3.10 (4.94)	0.0002 (0.0141)	10 000	38 956	0.153	255 431

Abbreviations: ICER, incremental cost-effectiveness ratio; NA, not applicable; QALY, quality-adjusted life-year.

**Figure 1.**
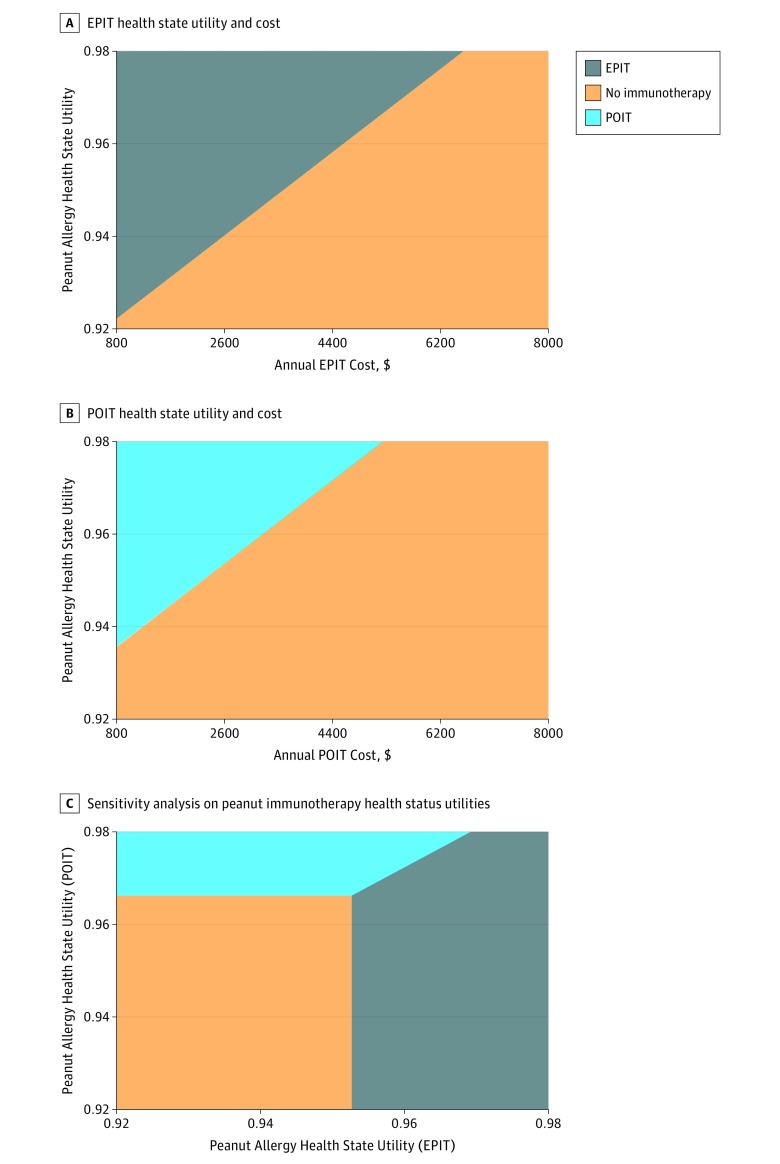
Association Between Immunotherapy Health State Utility and Cost A and B, Association of peanut allergy health state utility improvement with cost in epicutaneous immunotherapy (EPIT) (A) and peanut oral immunotherapy (POIT) (B) is shown for a net benefit at a willingness-to-pay threshold of $100 000 per quality-adjusted life-year, compared with a common baseline of no immunotherapy. C, Association of simultaneous peanut immunotherapy (POIT and EPIT) health state utility changes with cost-effectiveness is shown for a net benefit at a willingness-to-pay threshold of $100 000 per quality-adjusted life-year. Therapies remain cost-effective with greatest net monetary benefit within color-coded thresholds and ranges of health state utilities and costs.

When evaluating a value-based cost ceiling (at a WTP of $100 000/QALY vs no immunotherapy), EPIT and POIT were cost-effective at $1568 and $1235 per year, respectively (eFigure 2 in the Supplement). Further analyses of these therapies revealed an interaction between health state utility and the value-based cost ceiling, with the maximum value-based therapy cost of $6568 (EPIT) and $5235 (POIT) as health state utilities associated with therapies reached 0.98. The health state utility cost-effectiveness thresholds in the base case for EPIT and POIT were 0.953 and 0.966, respectively (Figure 1).

In the oral food challenge model, oral food challenge was performed (instead of assessing a serologic biomarker) after the first year to determine an eliciting dose of 300 mg (EPIT) or tolerated dose of 600 mg (POIT) of peanut protein to establish responder rates. Response rates to EPIT and POIT were modeled at 35.3% and 67.2%, respectively, without application of disutility for food challenge reactions. In the simulated model, participants who did not surpass these respective food challenge end points discontinued therapy, while those who surpassed these end points continued therapy. In this analysis, the ICER of each therapy was still greater than $100 000, referencing the common baseline of no immunotherapy (EPIT, $250 360 and POIT, $306 512). If disutility of reactive food challenges (−0.09) was modeled, EPIT was dominated (here, *dominated* is an economic term for a therapy that is associated with lower health benefit at a higher cost relative to another option), and the ICER of POIT was $417 635. Additional microsimulations were performed with POIT and assumed it to be associated with protection against 300 mg of peanut protein in 77% of participants after the first year. With an assumption of biomarker use detecting all of these participants (no need for food challenge), the ICER for POIT vs no therapy was $818 603. With food challenge instead of biomarker use after year 1 (without positive challenge disutility), a 77% response rate to POIT of at least 300 mg protection, and 600 mg protection in 67.2% of participants, the POIT ICER was $550 493.

**Figure 2.**
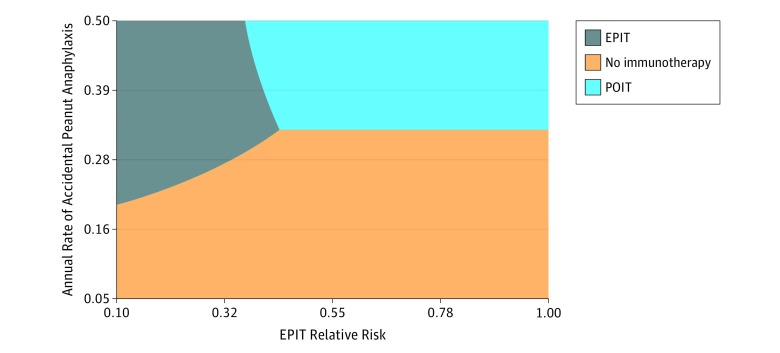
Three-Way Sensitivity Analysis of Epicutaneous Immunotherapy (EPIT) and Peanut Oral Immunotherapy (POIT) Protective Effect on Anaphylaxis Cost-effectiveness of therapies is shown across rates of accidental peanut anaphylaxis with EPIT relative risks of accidental anaphylaxis, at a POIT relative risk of 0.325.

In the base-case model, therapeutic benefit was associated with improvement in health state utility, which was in turn associated with perception of risk reduction of naturally occurring accidental severe allergic reactions. In a supplemental model, baseline risk of anaphylaxis was evaluated together with immunotherapy protection against severe accidental reactions (Figure 2). However, even when the annual rate of anaphylaxis was assumed to be 7% and EPIT and POIT provided a 50% risk reduction, therapies were not associated with a cost-effective ICER (EPIT, $172 683 and POIT, $195 370).

In simulations of durable SU to peanut allowing ad libitum dietary peanut consumption, participants achieving SU after 4 years transitioned off immunotherapy and continued to keep peanut in the diet without risk of reactions. If 19% of EPIT-treated children achieved SU after 4 years of treatment, then the therapy was cost-effective under base assumptions. After 4 years of POIT, if 30% achieved SU, this therapy provided greater net monetary benefit compared with EPIT or no immunotherapy (eFigure 3 in the Supplement). At a 50% SU rate after 4 years, the ICERs for EPIT and POIT were $16 886 and $20 403, respectively (mean [SD] episodes of anaphylaxis: EPIT, 0.98 [1.31] and POIT, 2.32 [3.87]). At a 75% SU rate after 4 years, both therapies dominated a no-therapy approach by producing savings in both cost and QALY. At a 7% annual base rate of anaphylaxis, microsimulations demonstrated EPIT was associated with fewer episodes of anaphylaxis than no immunotherapy at a 50% 4-year SU rate, or a 25% SU rate with a 0.5 relative risk of anaphylaxis while receiving therapy. Treatment with POIT was associated with fewer episodes of anaphylaxis than no therapy at a 75% 4-year SU rate (eTable 2 in the Supplement).

As POIT may be associated with higher front-end costs, we also evaluated an economic model in which the first-year cost of POIT was $5000 with a $1000 annual cost in subsequent years. Under these conditions, the mean (SD) cost of the POIT strategy over the model horizon was $141 773 ($21 859) and the ICER against no immunotherapy was $105 028. At a $10 000 first-year cost, then $400 per month thereafter, the POIT ICER was $366 329. A first-year cost of $7000 followed by $250 per month in subsequent years delivered an ICER of $346 241 over the base model horizon.^39^

### Additional Sensitivity Analyses

In sensitivity analyses, the ICERs of EPIT and POIT exceed $200 000 at additional model time horizons of 5, 10, 20, 40, and 60 years. Assuming incremental health state utility improvements did not occur until after the first year of therapy further increased the ICER of each therapy ($255 718 for EPIT and $286 100 for POIT). Use of the 80% estimated predictive biomarker value was not associated with cost-effectiveness for EPIT or POIT. Exclusion of biomarkers after the first year of therapy (assuming all participants benefit and continue receiving each therapy) was also not associated with cost-effectiveness (ICER against no treatment baseline: EPIT, $240 146 and POIT, $254 507). If 10% EPIT and 30% POIT response rates were modeled based on biomarker use, the ICER of the therapies against the common baseline was $265 619 (EPIT) and 298 783 (POIT). When we modeled the sensitivity of higher cost of hospitalization for an anaphylactic reaction, up to a cost of $20 000 (n = 10 000), the ICER for therapies continued to exceed $200 000/QALY (EPIT, $225 766 and POIT, $281 457). If a lower rate of EPIT-associated anaphylaxis (0.6%) was modeled after the first year,^4^ the EPIT ICER was $205 925. If a short time horizon was modeled to age 12 years (the age limit at which EPIT would be approved for starting), the ICER of EPIT was $244 392 and the ICER of POIT was $287 457. Microsimulation at very low rates of accidental anaphylaxis (0.5% per year) resulted in an ICER of $220 085 for EPIT and $269 133 for POIT.

**Figure 3.**
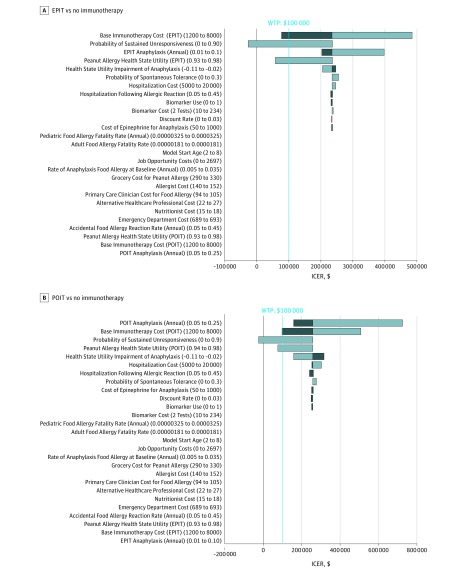
Tornado Diagrams of Deterministic Sensitivity Analyses A and B, Analyses shown for epicutaneous immunotherapy (EPIT) (A) and peanut oral immunotherapy (POIT) (B) against a common baseline of no immunotherapy. Light bars indicate values above and dark bars values below base-case assumptions. ICER indicates incremental cost-effectiveness ratio. Values in parentheses depict ranges across which deterministic sensitivity analyses were performed for each variable.

In probabilistic sensitivity analyses (n = 1000) neither EPIT or POIT were cost-effective at a WTP of $100 000 using base costs in any iteration (eFigure 4 in the Supplement). Evaluation of spontaneous tolerance rates did not create a more cost-effective model. At a 20% spontaneous tolerance rate over 16 years, the cost for peanut immunotherapy was $248 071/QALY (EPIT) and $269 253/QALY (POIT). When this spontaneous tolerance rate was assumed across immunotherapy health state utilities of 0.93 (EPIT) and 0.94 (POIT) improving to 0.98, the ceiling value-based cost of therapies was $1483 to $6483 (EPIT) and $1161 to $5161 (POIT). Deterministic sensitivity analyses on all variables revealed both therapies to be cost-effective across some thresholds of health state utility improvement, cost, and probability of SU (Figure 3).

Last, a highly idealized scenario was analyzed in a sensitivity analysis in which immunotherapy provided a 50% reduction to a 7% annual rate of anaphylaxis in association with a 0.98 health state utility for immunotherapy and 20% rate of 4-year SU. With these optimal circumstances, the ceiling value-based costs of EPIT and POIT were $9595 and $8297 per year, respectively.

## Discussion

This cost-effectiveness simulated analysis demonstrates that both EPIT and POIT may be cost-effective options, but under base-case assumptions the annual costs of each therapy could not exceed $1568 and $1235 (inclusive of clinician dose-escalation visits), respectively (at a WTP of $100 000/QALY). As this analysis predates approval of either product, it remains to be seen how much these products will actually cost. However, performing such analysis ahead of pricing or approval provides a view of the estimated potential value-based range of such a product and could enable more informed decision making.

Our analysis demonstrates 3 crucial levers affecting the cost-effectiveness and value of each peanut allergy therapy—health state utility improvement, risk reduction of anaphylaxis, and likelihood of achieving SU, at which point therapy could be discontinued. With more pronounced health state utility improvements, the value-based cost of each therapy could potentially reach $6568 (EPIT) and $5235 (POIT) annually. Additionally, evidence that either product produces a durable, lasting SU may increase their potential respective values over the base model, although the SU cost-effectiveness thresholds differ for each. For either therapy, if 4-year SU was obtainable for 75% of participants, who could then discontinue therapy, both forms of therapy dominated the no-therapy approach. As the therapies reliably offer protection against anaphylaxis, their cost-effectiveness may also become better leveraged. Commercial cost is clearly an important input, as is exemplified in a prior analysis^13^ of a related therapy, POIT combined with a probiotic, in which the ICER for this therapy vs no treatment was less than $3000 for a therapy using store-bought peanut flour acquired for less than $20.

The present understanding of patient-reported QoL changes under conditions of risk (utilities) for these therapies (and the understanding of the natural history of peanut allergy itself) is scant, but an important factor in QoL is likely the degree to which patients will perceive the benefit of the therapy. Such benefit depends on (1) the level of protection against and degree of risk reduction of allergic reactions, anaphylaxis, and fatalities; (2) the perceived anxiety reduction afforded for patients and families; and (3) the extent to which patients can make dietary changes as a result of successful therapy. Recent qualitative work identified that families value both the reduced risk that an accidental reaction will occur and also reduced consequences of such an exposure (ie, prevention of fatality and anaphylaxis).^8^ Fortunately, peanut allergy–associated fatality is rare, and cost-effectiveness based solely on this outcome would be difficult to attain.^12,40^

A particular advantage to this analysis is the inclusion of estimated biomarkers to measure progress or predicted probability of response vs nonresponse, an important consideration^25^ given issues surrounding the safety, desirability, and practicality of conducting food challenges as the sole measure of therapeutic progress. Identifying patients who respond poorly prevents continuing an expensive, ineffective treatment associated with potentially significant risks compared with no therapy at all. To account for uncertainties associated with biomarker use, our analysis included evaluations of therapies using food challenge instead of biomarkers and models without biomarkers. Still, further work is needed to better understand the predictive capacities of food immunotherapy biomarkers.

### Limitations

There are limitations of this analysis, some of which were addressed in broad sensitivity analyses. First, this analysis is based on not-yet-approved products, and these models will require iterative adjustment as more information becomes available. Second, we recognize that a food allergy–specific study of WTP for treatments with these attributes, as well as establishment of health utility in both a treated and untreated food-allergic population, are needed to help clarify assumptions. It is uncertain how, in the context of 2 very specific treatments, WTP and health utility may differ from present values.^33^ Third, our inputs and assumptions are based on the 2 phase 3 trial outcomes for each product, one of which was negative per the prespecified statistical analysis plan as the lower boundary of the treatment group effect crossed a prespecified limit of 15% (set to infer a margin of super-superiority vs placebo to approximate clinical significance), despite a statistically significant between-group difference favoring treatment.^3,5^ This predisposes the study to potential information ascertainment bias and could limit generalizability, given no trial replication or real-world experience demonstrating certain outcomes will not shift outside of a clinical trial. Fourth, there are no long-term outcome data available to better inform the degree to which dropout or achieving SU may occur, or to be able to model preliminary additional rates of adverse events occurring in POIT after 12 months of therapy (as was available for EPIT).^7^ Fifth, the degree to which a caregiver preference for a particular product or attribute profile of either therapy is unknown at present, although this is being studied. Sixth, we could not model (given no data regarding this from either phase 3 trial) evidence from Israeli POIT studies indicating that many patients receiving POIT develop peanut aversion even when desensitized, which limited treatment compliance.^41^ Seventh, we did not model alternative potential paradigms that have been proposed for establishing value-based care with more mature products, already on the market, with a price and reimbursement set by managed care.^42^

## Conclusions

Both EPIT and POIT are peanut allergy treatment options on the horizon. Both therapies may be cost-effective under some assumptions, although in the base-case analysis in this study, these eclipse the threshold of $100 000/QALY used in the United States. However, these estimates were sensitive to particular levers, and a cost-effectiveness ceiling was estimated for each product. Understanding what these levers are is important prior to product approval and launch and could be an advantage to make pricing decisions more efficient. Further research is needed to understand the degree of health state utility improvement associated with each therapy, and longer-term commercial data will continue to inform health and economic analyses.

## Notes

Supplement. **eTable 1.** Glossary of Terms **eTable 2. **Total Episodes of Anaphylaxis Over the Model Horizon **eFigure 1. **Decision Model **eFigure 2. **Value-Based Costing of Immunotherapy **eFigure 3. **Analysis of Sustained Unresponsiveness at 4 Years **eFigure 4. **Cost-effectiveness Acceptability Curve
