# Supplementary material for: Estimation of Health and Economic Benefits of Commercial Peanut Immunotherapy Products: A Cost-effectiveness Analysis
Source: JAMA Netw Open. 2019 May 3;2(5):e193242. doi: 10.1001/jamanetworkopen.2019.3242 (PMC6503512; doi:10.1001/jamanetworkopen.2019.3242)
Supplement: Supplement. — eTable 1. Glossary of Terms eTable 2. Total Episodes of Anaphylaxis Over the Model Horizon eFigure 1. Decision Model eFigure 2. Value-Based Costing of Immunotherapy eFigure 3. Analysis of Sustained Unresponsiveness at 4 Years eFigure 4. Cost-effectiveness Acceptability Curve [file jamanetwopen-2-e193242-s001.pdf]

Supplementary Online Content

Shaker M, Greenhawt M. Estimation of health and economic benefits of commercial peanut immunotherapy products: a cost-effectiveness analysis. *JAMA Netw Open*. 2019;2(5):e193242. doi:10.1001/jamanetworkopen.2019.3242

eTable 1. Glossary of Terms
eTable 2. Total Episodes of Anaphylaxis Over the Model Horizon
eFigure 1. Decision Model
eFigure 2. Value-Based Costing of Immunotherapy
eFigure 3. Analysis of Sustained Unresponsiveness at 4 Years
eFigure 4. Cost-effectiveness Acceptability Curve

This supplementary material has been provided by the authors to give readers additional information about their work.

© 2019 Shaker M et al. *JAMA Network Open.*

eTable 1: Glossary of Terms[a]

Terms Commonly Used in Cost-Effectiveness Analyses
**Incremental Cost-Effectiveness Ratio:** a ratio comparing the costs and benefits of strategy A with the costs and benefits of strategy B to help place a perspective of the relative value of each strategy.
**Cost-effectiveness analysis:** a complex valuation that grades both health outcomes and economic benefits of a particular aspect of clinical care, using inputs that integrate the broad medical evidence surrounding a concept, the impact on a patient with disease, and the costs and impact of those costs (which can be explored at a patient, payer, or society level), explored over a particular time horizon of the condition.
**Societal perspective:** a perspective from which to model a cost-effectiveness analysis which considers everyone who may be affected by a specific intervention, where costs may be shared between patients, employers, payers, and society, which does not depend on who pays the cost and who receives the benefit.
**Payer perspective:** a perspective from which to model a cost-effectiveness analysis which considers only the direct medical costs incurred by payers as well as the direct out of pocket costs that patients may pay.
**Markov models:** specific simulations widely used in healthcare that allow transitions between health states (e.g., sick, well, dead) to replicate clinical events that may happen over the course of experiencing a disease in a lifetime. Evaluation of models through probabilistic sensitivity Markov microsimulation allows sampling of distributions of probabilities, costs, and other events to introduce additional second-order stochastic variation involving the interactions of multiple variables and probabilities.
**Health utility:** also defined as a health state utility. This is a patient preference for a particular health state, on a probability scale with 0 representing death and 1 representing perfect health. Health utility can be assessed in a direct manner, through questions that asks patients to rate their health along a visual analog scale in relation to either a time trade off or standard gamble scenario asking the patient to make a determination of how much time they would trade to have perfect health, or through scenarios that assess the willingness to gamble varying degrees of health to seek a cure or treatment for a disease.
**Quality Adjusted Life Year (QALY):** a scaled value of a hypothetical health state which incorporates health utility in assessing how effective an intervention may be in terms of demonstrating both quality of life and quantity of life in a time period with a disease. QALY are frequently used because they combine morbidity and mortality in a single standardized measure that can be used across health and disease states, given this is valuing both duration of life and the quality of that life within a disease in a fixed time unit. Determining QALY is a specific decision analysis method is often referred to as cost-utility (or health utility) analysis, because QALY measures are derived from health state utilities reported by populations, which represent patient preferences for one particular health state over another under conditions of risk.
**Sensitivity analyses:** evaluations within cost-effectiveness analyses which effectively re-analyze specific questions to explore the stability of conclusions over a range of plausible assumptions and identify potential levers and thresholds at which outcomes and conclusions change.
**Dominance:** dominant therapies refers to a health economic state for a particular therapy which, in comparison to one or more alternative therapies, produces lower costs and greater

© 2019 Shaker M et al. *JAMA Network Open.*

benefits when compared to another. A dominated therapy would produce higher costs and lower benefits.
**Net monetary benefit:** a summary statistic that represents the value of an intervention in monetary terms when a willingness-to-pay threshold for a unit of benefit (for example a measure of health outcome or QALY) is known. The use of NMB scales both health outcomes and use of resources to costs, with the result that comparisons without the use of ratios (such as in ICER). NMB is calculated as (incremental benefit x threshold) – incremental cost. Incremental NMB measures the difference in NMB between alternative interventions, a positive incremental NMB indicating that the intervention is cost-effective compared with the alterative at the given willingness-to-pay threshold. In this case the cost to derive the benefit is less than the maximum amount that the decision-maker would be willing to pay for this benefit.
**Willingness to pay:** this has a two-fold application but similar definition in that it represents the threshold that a person or a payer is willing to pay for a given therapy. At the patient level, this can represent how much direct out of pocket costs a patient will pay for a therapy. At a payer level, this can represent a currency threshold/limit (e.g. dollars, pounds, yen, etc,) per QALY in value delivered (e.g., cost-effectiveness) that a therapy must meet or exceed.
**Deterministic sensitivity analysis:** a sensitivity analysis in which one or more parameters are manually changed based on a pre-determined range (e.g. like across a 95% CI) to see how the change affects the outcomes, as a way to model uncertainty. The results of deterministic sensitivity analysis are usually expressed as line graphs or bar charts, such as a tornado diagram of bar graphs representing univariate sensitivity analyses for a wide range of input values, ordered according to the extent (spread) of variation of the resulting model output value (with the widest variation on top).
**Tornado diagram:** tornado diagrams are used to present the result of multiple univariate sensitivity analyses on a single graph. Each analysis is summarized using a horizontal bar which represents the variation in the model output (usually an ICER) around a central value (corresponding to the base case analysis) as the relevant parameter is varied between two plausible but extreme values. The horizontal bars are ordered so that with those with the greatest spread (i.e. parameters to which the model output is most sensitive) come at the top of the diagram, and those with the lowest spread at the bottom. The resulting diagram of stacked horizontal bars has a distinctive tornado shape. Tornado diagrams are used to help the reviewer assess which of the model's parameters have the greatest influence on its results.
**Value based pricing:** a pricing model for therapy where the reimbursement price of therapy depends on the outcomes that the therapy produces, where better outcomes can justify a higher value-based price.

[a]source: York Health Economics Consortium; 2016. https://www.yhec.co.uk/glossary

eTable 2: Total episodes of anaphylaxis over the model horizon.

	Total Episodes of Anaphylaxis Over the Model Horizon		
**Rate of SU**	**No Immunotherapy**	**EPIT**	**POIT**
	1% annual base rate of accidental severe reactions		
**0% SU**	0.72 (SD 0.85)	1.33 (SD 1.55)	3.83 (SD 5.02)
0.5 relative risk		1.23 (SD 1.42)	3.73 (SD 4.84)
0.2 relative risk		1.21 (SD 1.38)	3.65 (SD 4.78)
**25% SU**		1.17 (SD 1.44)	3.04 (SD 4.49)
0.5 relative risk		1.10 (SD 1.33)	2.95 (SD 4.37)
0.2 relative risk		1.04 (SD 1.25)	2.86 (SD 4.21)
**50% SU**		0.98 (SD, 1.31)	2.32 (SD, 3.87)
0.5 relative risk		0.92 (SD 1.20)	2.25 (SD 3.69)
0.2 relative risk		0.90 (SD 1.16)	2.18 (SD 3.56)
**75% SU**		0.81 (SD 1.12)	1.58 (SD 2.87)
0.5 relative risk		0.79 (SD 1.06)	1.52 (SD 2.74)
0.2 relative risk		0.76 (SD 1.01)	1.51 (SD 2.71)
	7% annual base rate of accidental severe reactions		
**0% SU**	5.06 (SD 2.34)	5.64 (SD 2.69)	8.04 (SD 5.55)
0.5 relative risk		5.07 (SD 2.35)	7.20 (SD 4.42)
0.2 relative risk		4.62 (SD 2.33)	6.87 (SD 3.91)
**25% SU**		5.19 (SD 2.81)	7.00 (SD 5.21)
0.5 relative risk		4.71 (SD 2.50)	6.45 (SD 4.39)
0.2 relative risk		4.46 (SD2.51)	6.12 (SD 3.92)
**50% SU**		4.82 (SD 2.93)	6.09 (SD 4.83)
0.5 relative risk		4.41 (SD 2.72)	5.62 (SD 4.16)
0.2 relative risk		4.21 (SD 2.70)	5.43 (SD 3.77)
**75% SU**		4.40 (SD2.88)	5.00 (SD 4.03)
0.5 relative risk		4.23 (SD 2.79)	4.85 (SD 3.62)
0.2 relative risk		4.08 (SD 2.77)	4.71 (SD 3.41)

Episodes of anaphylaxis shown by rates of sustained tolerance at 4 years and relative risks of severe accidental reactions to peanut.

© 2019 Shaker M et al. *JAMA Network Open.*

eFigure 1: Decision model.

Panel A:

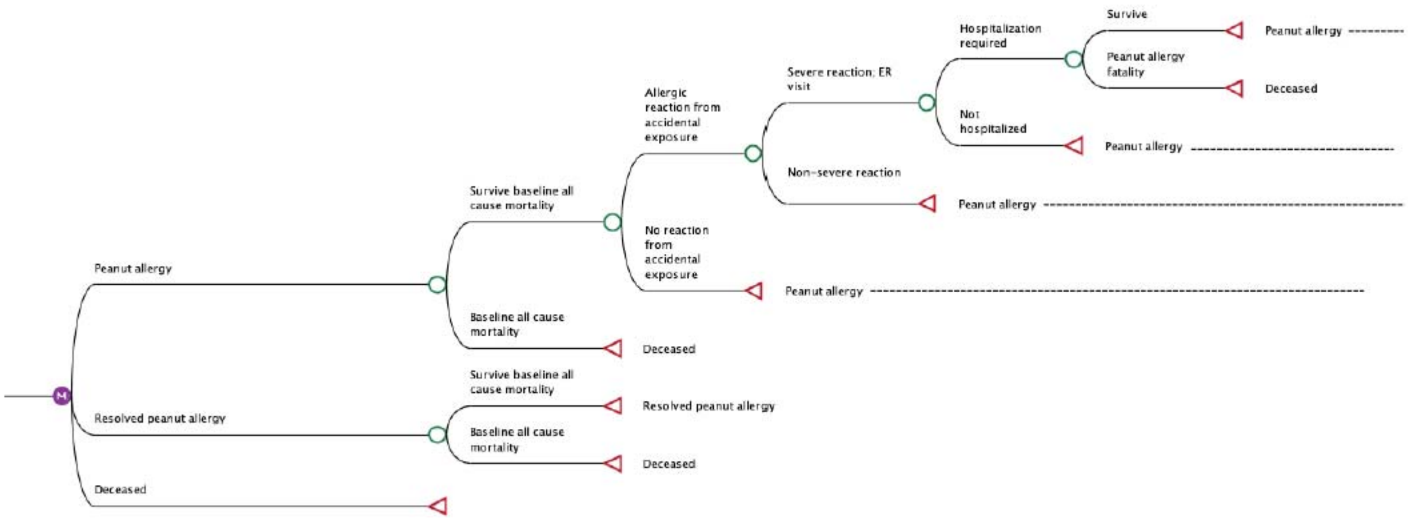

© 2019 Shaker M et al. *JAMA Network Open.*

Panel B:

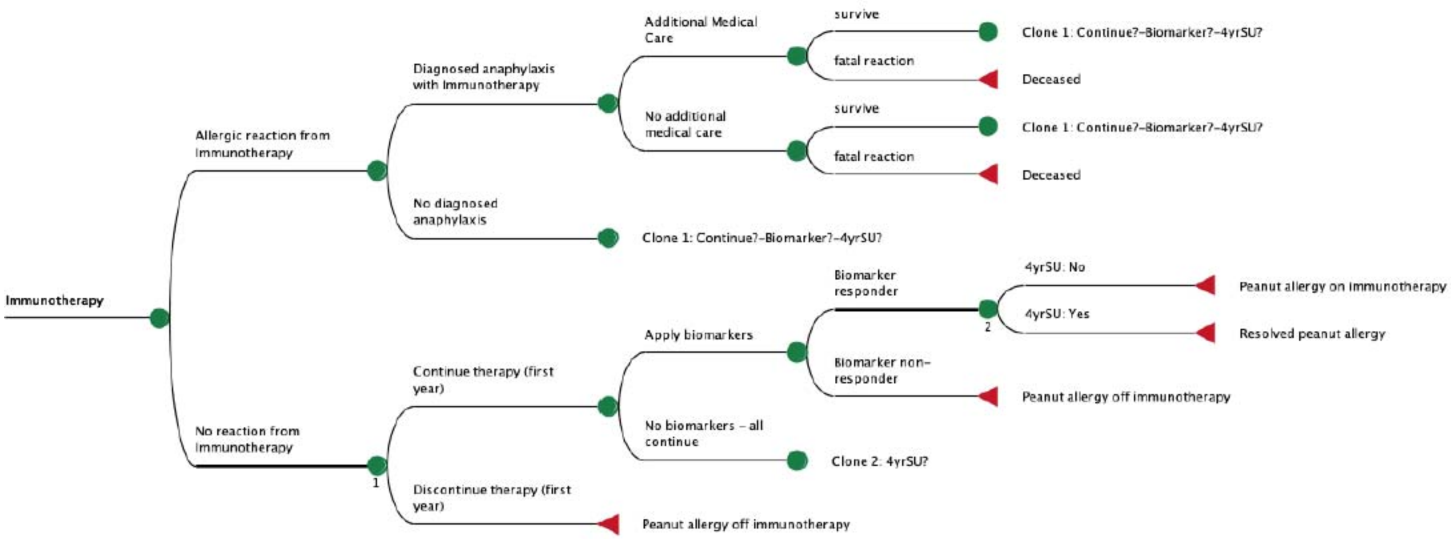

Legend:

Panel A: Peanut allergy health state. Subjects in all arms experienced probabilities and costs of peanut allergy. Subjects in EPIT or POIT treatment also experienced probabilities associated with EPIT or POIT while accumulating health state utility benefits associated with these therapies.

Panel B: Decision model of immunotherapy health states. Subjects in the EPIT and POIT treatment arms experienced probabilities associated with iterative dichotomous outcomes under unique probabilities and costs associated with each therapy.

© 2019 Shaker M et al. *JAMA Network Open.*

eFigure 2. Value based costing of immunotherapy.

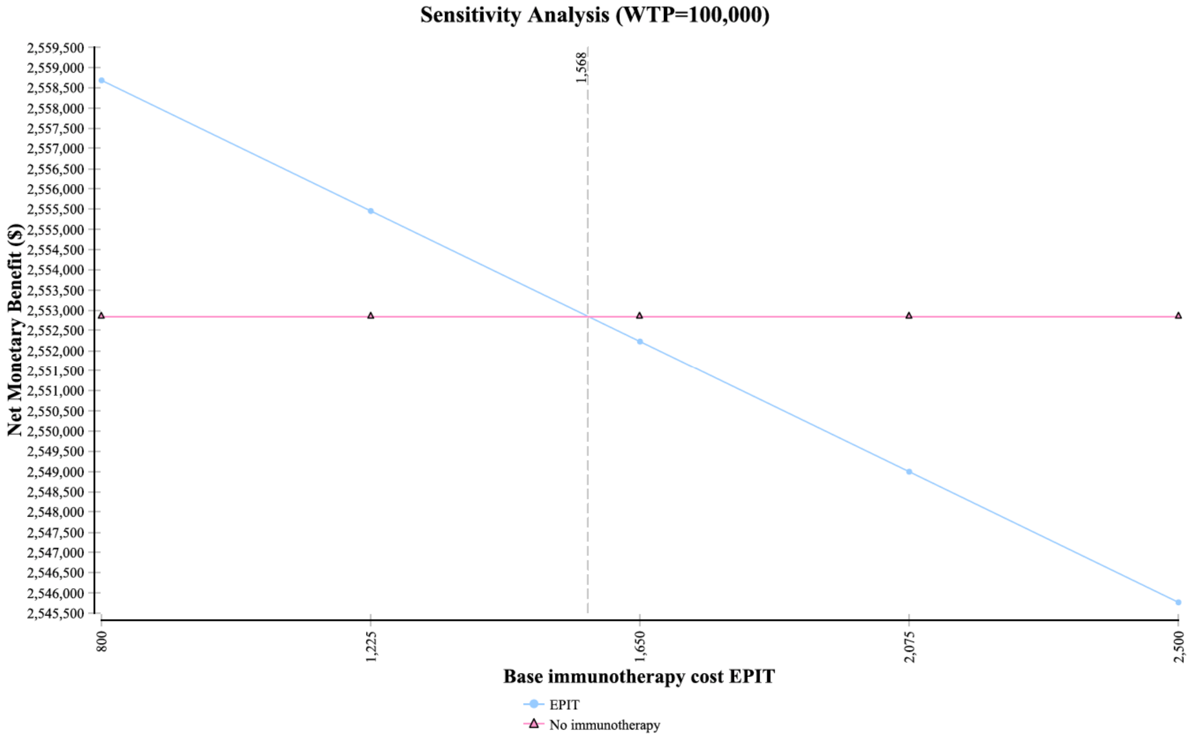

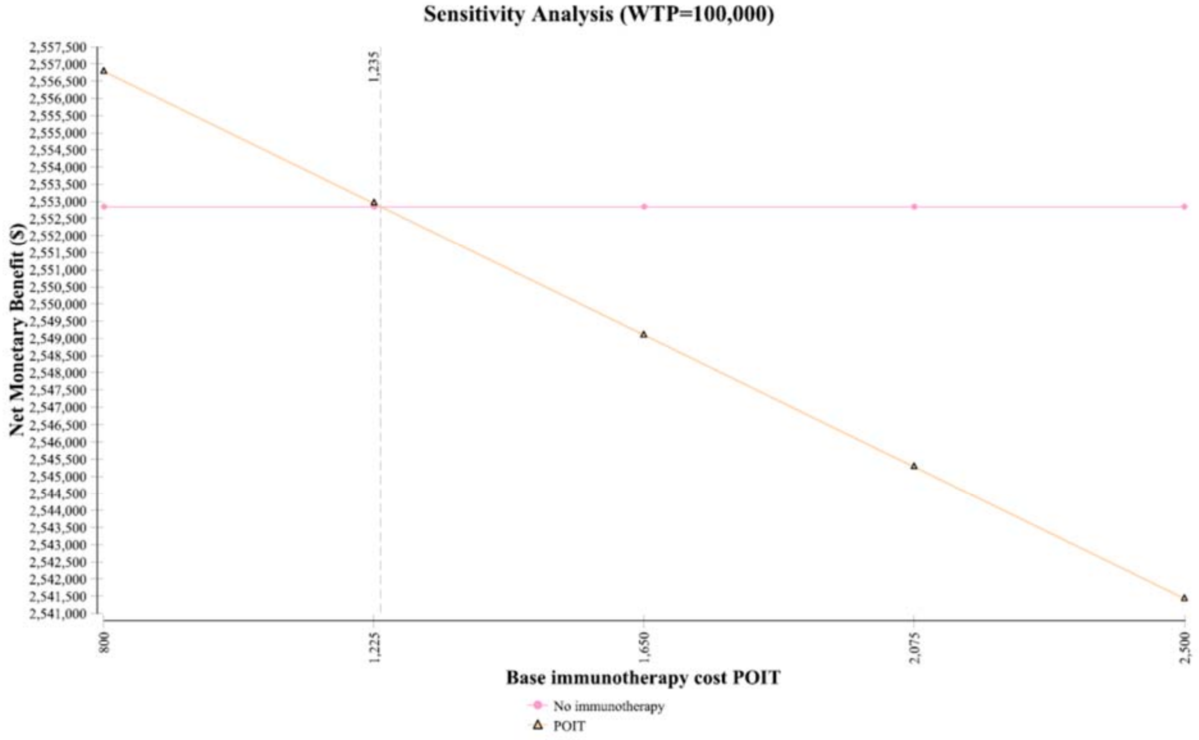

Legend: Value-based cost-ceiling EPIT (upper) and POIT (lower) under base-case assumptions.

© 2019 Shaker M et al. *JAMA Network Open.*

eFigure 3. Analysis of Sustained Unresponsiveness at 4 years.

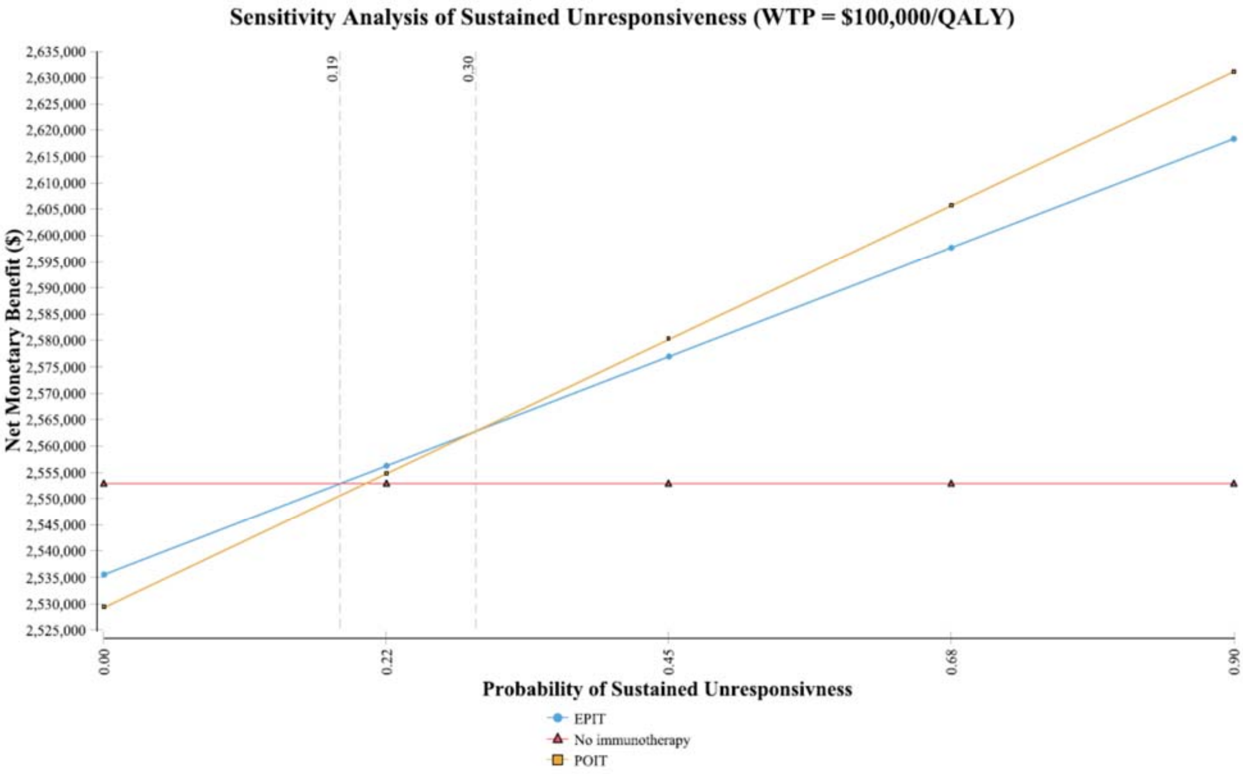

Legend: Net monetary benefit (NMB) of peanut immunotherapies and of no therapy shown across rates of sustained unresponsiveness after 4 years of therapy. At an SU rate of 19% EPIT was associated with the greatest NMB, while at rates above 30% NMB was highest for POIT.

© 2019 Shaker M et al. *JAMA Network Open.*

eFigure 4. Cost-effectiveness Acceptability Curve.

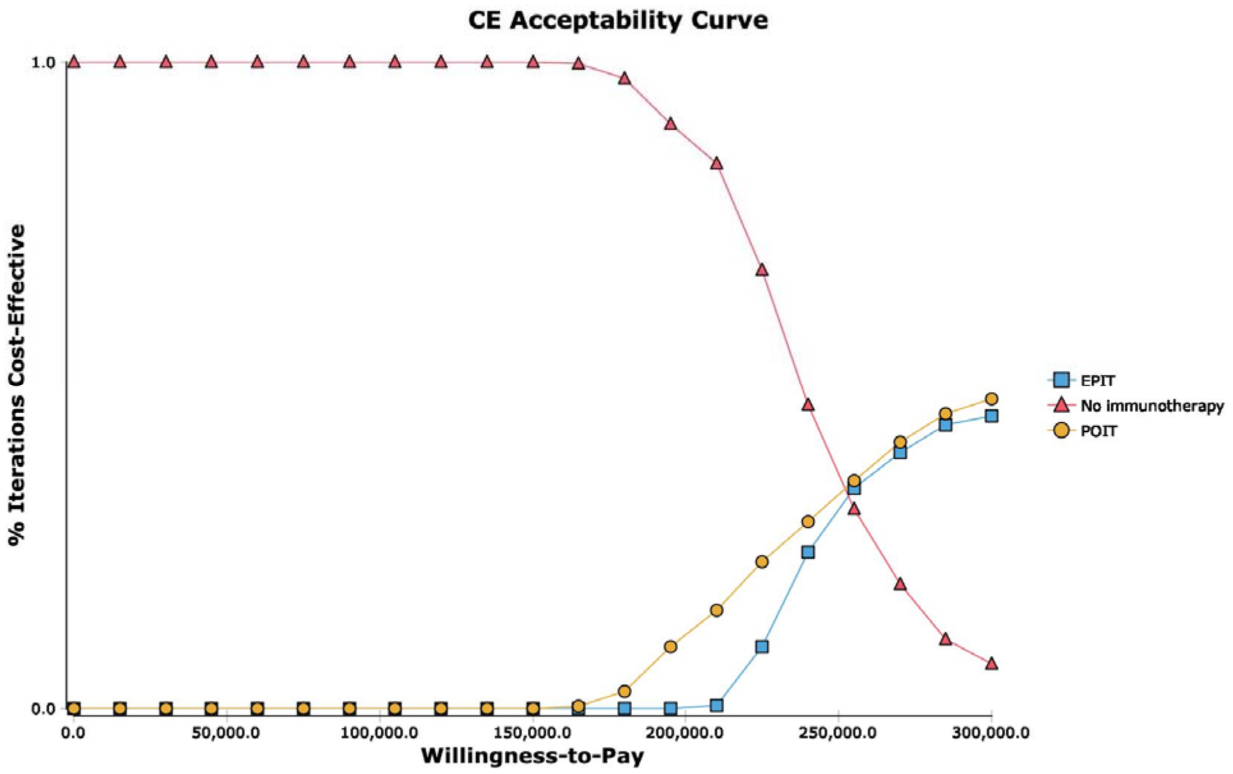

Legend: In probabilistic sensitivity analyses (n=1,000) neither EPIT or POIT were cost effective at a WTP of $100,000 using base costs.

© 2019 Shaker M et al. *JAMA Network Open.*
